# Optimal Dual RNA-Seq Mapping for Accurate Pathogen Detection in Complex Eukaryotic Hosts

**DOI:** 10.21769/BioProtoc.5182

**Published:** 2025-02-05

**Authors:** Infanta Saleth Teresa Eden M., Umashankar Vetrivel

**Affiliations:** Department of Virology and Biotechnology, Bioinformatics Division, Indian Council for Medical Research-National Institute for Research in Tuberculosis (ICMR-NIRT), Chennai, India

**Keywords:** Dual RNA-Seq, Host–pathogen interactions, Host-first mapping, Pathogen-first mapping, Misalignment of pathogen reads

## Abstract

Dual RNA-Seq technology has significantly advanced the study of biological interactions between two organisms by allowing parallel transcriptomic analysis. Existing analysis methods employ various combinations of open-source bioinformatics tools to process dual RNA-Seq data. Upon reviewing these methods, we intend to explore crucial criteria for selecting standard tools and methods, especially focusing on critical steps such as trimming and mapping reads to the reference genome. In order to validate the different combinatorial approaches, we performed benchmarking using top-ranking tools and a publicly available dual RNA-Seq Sequence Read Archive (SRA) dataset. An important observation while evaluating the mapping approach is that when the adapter trimmed reads are first mapped to the pathogen genome, more reads align to the pathogen genome than the unmapped reads derived from the traditional host-first mapping approach. This mapping method prevents the misalignment of pathogen reads to the host genome due to their shorter length. In this way, the pathogenic read information found at lesser proportions in a complex eukaryotic dataset is precisely obtained. This protocol presents a comprehensive comparison of these possible approaches, resulting in a robust unified standard methodology.

Key features

• Benchmarking of top-ranking software for quality control, adapter trimming, and read mapping.

• Emphasizes the importance of read mapping criteria for dual RNA-Seq datasets: (i) high count of uniquely host mapped reads, (ii) low count of host multi-mapped reads, and (iii) high count of unmapped reads belonging to pathogens.

• Elaborates the best mapping approach to precisely extract the pathogen reads as these get captured comparatively less in dual RNA-Seq datasets.

Graphical overview

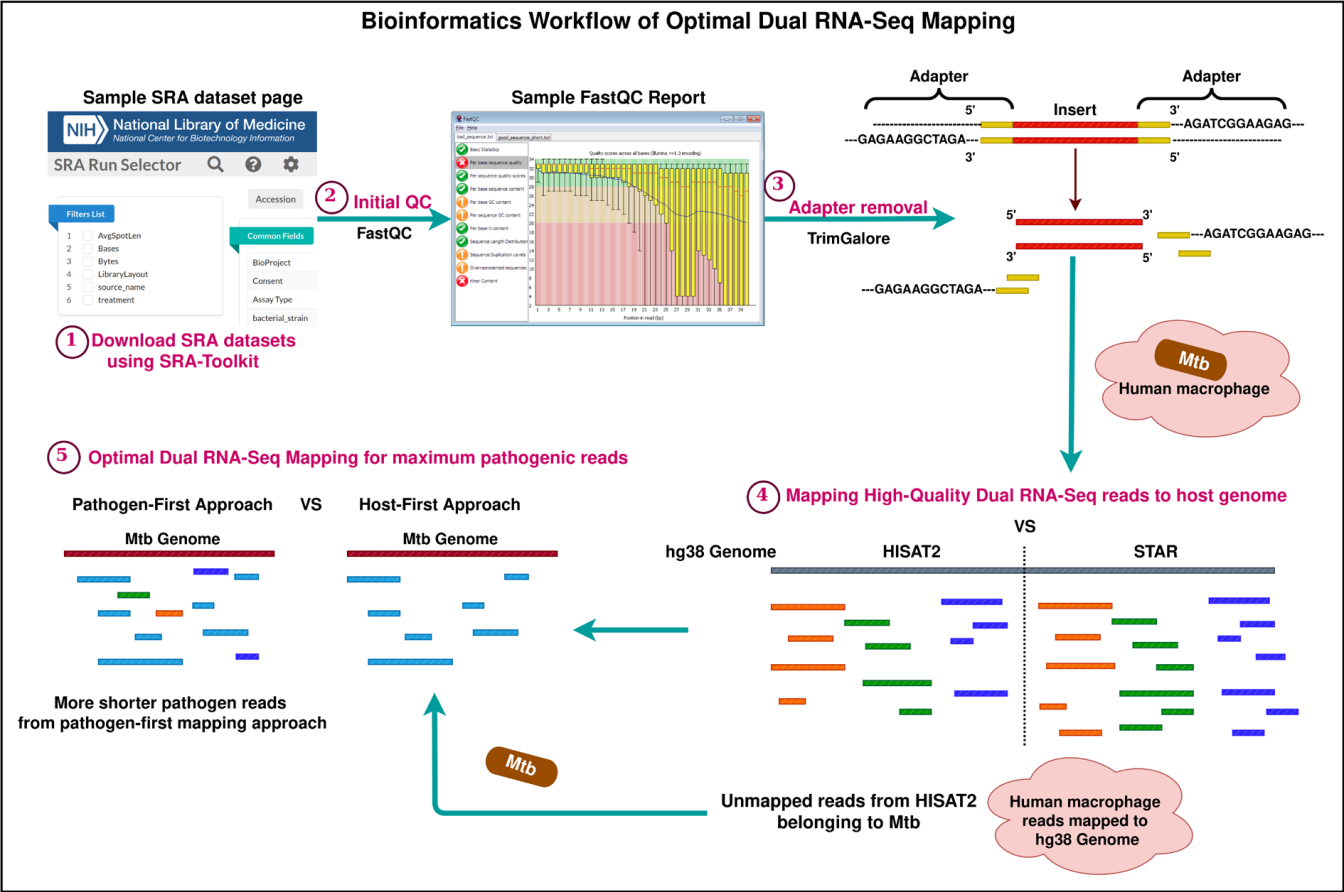

## Background

Dual RNA sequencing is a powerful tool to precisely investigate the complete gene expression profile of two actively interacting organisms. When a pathogen infects a host, both develop adaptation mechanisms by revealing a series of changes at molecular levels. These biological changes are interrelated and can be effectively elucidated by analyzing the whole transcriptome data of the host–pathogen interaction. Until 2012, transcriptomics sequencing of infectious diseases was limited to separately capturing the cascade of biological events in either the host or the pathogen [1]. This allows us to study gene expression changes only from a linear perspective. Over the years, insightful research in transcriptomics analysis has encouraged researchers across the globe to devise the concept of extracting and sequencing whole RNA from the infection site. This idea will provide a broader scope for unraveling the role of smaller molecules and studying the biological changes in organisms at different stages of interaction. Compared to conventional RNA sequencing technology, dual RNA sequencing caters to a sufficient amount of host and pathogen RNA and other small RNA molecules (microRNA, long non-coding RNA, and other non-coding RNA) when pathogen-infected samples are subjected to the well-established and published methods of RNA extraction and sequencing procedures [2–5].

Dual sequencing data generated as reads or fragments comprises information on both the host and the pathogen. The infected host cells may not always reflect pathogen-related data in enormous quantities. Therefore, a detail-oriented analysis is necessary to capture the minimally found essential information on host–pathogen interplay. Several bioinformatics analysis methods and tools are available to explore the complex datasets involving eukaryotic and prokaryotic reads [6–8]. Most of these methods suggest a sequential mapping approach to extract host and pathogen reads. Some studies recommend a combined mapping approach where the reference genomes of the host and pathogen are concatenated, indexed, and used as a single reference [9,10]. As far as quality control and adapter removal are concerned, standard methods for read mapping have been receiving greater attention as pathogen data is found in lesser quantities. Espindula et al. [10] conducted studies on different infection models and showed that other alternative mapping approaches outperform the traditional host-first mapping approach. When trimmed reads are mapped first to the host reference, there are high chances of pathogen read mismapping due to its shorter read length. To avoid this, alternative mapping approaches were introduced, and this bio-protocol emphasizes one such mapping technique—the pathogen-first mapping approach—in detail. A comparison of mapping results proved that most of the pathogen reads have been restored in the pathogen-first mapping approach. The results obtained using this approach are now based on a higher confidence scale and can be used for further processing.

This protocol is presented with the aim of demonstrating a standardized bioinformatics procedure for productive mapping. In this protocol, human monocyte-derived macrophages (HMDMs) infected with *Mycobacterium tuberculosis* (Mtb) were used as a dual RNA-Seq model. Apart from humans infected with Mtb, other host and pathogen models can still be used in dual RNA-Seq experiments. For example, other dual RNA-Seq datasets are publicly available in Gene Expression Omnibus (GEO); one such dataset is *Triticum aestivum* infected with *Fusarium graminearum* (Accession: SRP439529; GEO: GSE233409). Dual RNA-Seq test dataset of human–Mtb was downloaded from Sequence Read Archive (SRA), National Centre for Biotechnology Information (Accession: SRP359986 [11]). This study primarily focuses on a compound that restricts *Mycobacterium tuberculosis* from catabolizing cholesterol by binding with iron. The data quality control before and after read trimming was performed using FastQC. The FastQC tool can be accessed online at https://www.bioinformatics.babraham.ac.uk/projects/fastqc/. For trimming low-quality bases and adapter removal, benchmarking was performed using the topmost adapter trimming software, namely fastp [12] and Trim-Galore (https://github.com/FelixKrueger/TrimGalore). Following this, the topmost splice-aware alignment tools STAR [13] and HISAT2 [14] were also benchmarked to obtain optimal results. The most important part of the analysis is to apply the best mapping method that serves the experimental purpose of dual RNA-Seq analysis. After mapping the reads to their respective genomes, the reads mapping to genes were quantified using featureCounts [15]. Other downstream analysis methods, which include read count normalization, differential expression analysis, gene ontology, and pathway enrichment, are not demonstrated here, as the key aim of this protocol is to highlight the crucial preparatory steps like quality control, adapter removal, and read mapping in a descriptive manner.

## Software and datasets

1. Data

Dual RNA-Seq datasets are publicly available on NCBI Sequence Read Archive (SRA) and can be downloaded and analyzed for learning purposes. For this protocol, a dataset from SRA (accession: SRP359986, Gene Expression Omnibus datasets; GEO: GSE196816) was downloaded and utilized.

2. Bioinformatics tools (all tools were installed using conda)

• SRA-Toolkit (version 3.1.0) includes tools like prefetch and fasterq-dump for fetching SRA datasets and extracting individual fastq files.

• FastQC (version 0.12.1). For assessing the quality of fastq reads before and after trimming.

• MultiQC (version 1.19). For integrating results into interactive visualization reports throughout the analysis.

• TrimGalore (version 0.6.10) and Cutadapt (version 4.6). For quality-trimming bases from reads, automatic adapter detection and removal, and filtering reads based on lengths.

• HISAT2 (version 2.2.1). For indexing the reference genome and mapping trimmed high-quality reads to the reference genome of eukaryotes.

• BWA (version 0.7.17-r1188). For indexing the reference genome and mapping trimmed high-quality reads to the reference genome of prokaryotes.

• SAMtools (version 1.19). For converting huge files from mapping results (.sam) into binary formatted .bam files enabling easy processing, to sort reads with their mate pairs, and to check the statistical distribution of reads after mapping.

• Bedtools (version v2.31.1). For extracting the interleaved reads inside .bam files into paired-end separate fastq files.

• featureCounts from Subread package (version v2.0.6). For quantifying all reads mapped to genomic coordinates using annotation feature file(.gtf/.gff3) of the reference genome.

3. Platform used: Linux, Ubuntu

• CPU: Architecture, 64 bit; 24 cores, 96 threads

• Memory: 512 GB RAM

*Note: The threads/cores mentioned in each step of the analysis need to be modified by users as per the computational resources available.*

## Procedure

**Pseudocode for the steps used in the analysis:**

START OF ANALYSIS

# Step 1: Downloading SRA Datasets and Preparation of raw fastq files

DOWNLOAD the list of SRA accession ids

CREATE a file with SRA accession list

USE ‘prefetch’ command on the SRA list to download data files

USE ‘fasterq-dump’ command to extract fastq files from the downloaded data files

USE ‘gzip’ command to compress extracted fastq files in .fastq.gz format

# Step 2: Initial Quality Control of raw fastq files

USE ‘fastqc’ command to check quality of raw data

USE ‘multiqc’ to create consolidated QC report of raw data

# Step 3: Data cleaning and Final Quality Control of trimmed data:

USE ‘trimgalore’ command to trim adapter reads and low-quality bases from raw reads

USE ‘fastqc’ command to check quality of adapter-trimmed reads

USE ‘multiqc’ command to create consolidated QC report of adapter-trimmed reads

# Step 4: Mapping high-quality fastq reads to the reference genome:

CREATE the hg38 reference genome index using ‘hisat2’

CREATE the Mtb reference genome index using ‘bwa’

USE ‘bwa’ to map the adapter-trimmed reads to Mtb genome index

USE ‘samtools’ to extract the unmapped reads from the generated .bam files

USE ‘bedtools bamtofastq’ to convert .bam files to .fastq files

USE ‘hisat2’ to map the unmapped reads to hg38 genome index

# Step 5: Quantification of reads mapped to genomic features:

USE ‘featurecounts’ on the mapped .bam files to count reads belonging to transcripts/genes/exons/

# Step 6: Downstream transcriptome analysis:

USE the readcounts table to perform gene expression analysis using statistical methods like DESeq2, edgeR or Cufflinks-Cuffdiff

USE tools and databases like ‘BINGO’, ‘CytoHubba’, ‘DAVID’, to identify ontologies and pathways of differentially expressed genes

USE homology search tools like ‘BLAST’ to annotate the differentially expressed genes

END OF ANALYSIS

**A. Downloading SRA datasets and preparation of raw fastq files**

The sequence datasets used were obtained from NCBI SRA from a dual RNA sequencing study conducted by Theriault et al. [11], where they identified a compound that restricts Mtb from catabolizing cholesterol by binding with iron. In the study, HMDMs were infected with Mtb, and the infected cells were exposed to the following drug treatments: 5 μg/mL ethambutol, 67.5 ng/mL isoniazid, 10 μM mCLB073, 10 μM sAEL057, and dimethyl sulfoxide (DMSO; untreated). For demonstrative purposes, we chose to work with DMSO (untreated) for the control group, and 10 μM mCLB073 and 10 μM sAEL057 for the treatment groups (Table 1). The dataset included both paired-end and single-end read samples; hence, the protocol demonstrates processing both types of libraries in each step.

Table 1 lists the SRR IDs, library type, and treatment given for all samples. All nine sample runs were downloaded from SRA using the SRR run IDs.

**Table 1.** Sample information

SRR run IDs	Library	Treatment (Mtb-infected human macrophages)
SRR18042662	Paired	DMSO; untreated control
SRR18042663	Single	DMSO; untreated control
SRR18042664	Single	DMSO; untreated control
SRR18042665	Paired	10 μM mCLB073
SRR18042666	Single	10 μM mCLB073
SRR18042667	Single	10 μM mCLB073
SRR18042668	Paired	10 μM sAEL057
SRR18042669	Single	10 μM sAEL057
SRR18042670	Single	10 μM sAEL057

The sequence reads data were downloaded using the prefetch tool from SRA-Toolkit (version 3.1.0). SRA-Toolkit can be downloaded from https://trace.ncbi.nlm.nih.gov/Traces/sra/sra.cgi?view=software. The paired-end and single-end reads were extracted from the downloads using fasterq-dump from SRA-Toolkit.

**Code snippet for downloading RNA-Seq datasets from NCBI-SRA:**

$ prefetch SRR18042662 SRR18042663 SRR18042664 SRR18042665 SRR18042666 SRR18042667 SRR18042668 SRR18042669 SRR18042670

$ fasterq-dump --threads 50 SRR18042662 SRR18042663 SRR18042664 SRR18042665 SRR18042666 SRR18042667 SRR18042668 SRR18042669 SRR18042670

Subsequently, the paired and single-end fastq files extracted from SRA downloads were compressed to .fastq.gz format using gzip. The compressed fastq files are the raw reads that will be utilized for the downstream analysis.

**B. Initial quality control using FastQC**

**Figure 1.**
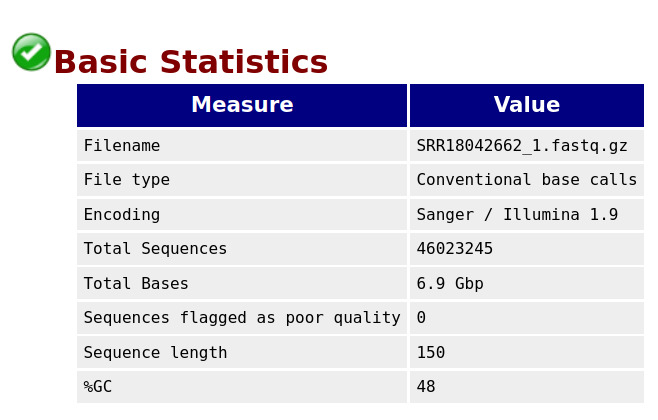
Initial QC: Basic statistics for sample SRR18042662

**Figure 2.**
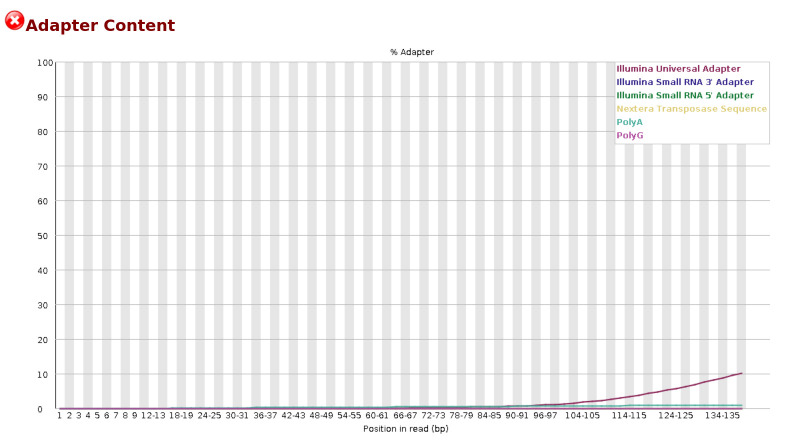
Initial QC: Adapter content before trimming for the sample SRR18042662

The quality of reads and bases from the raw FASTQ files were assessed using FastQC. This tool checks the number of reads and their quality, the number of bases and their quality, the presence of adapters, and other statistics such as read length distribution and GC content. FastQC generates separate visualization reports for forward and reverse-read files in HTML format. The HTML files from all samples are then consolidated into a single interactive visualization report using MultiQC [16]. Figures 1 and 2 represent the basic statistics and adapter content before trimming of SRR18042662 sample dataset using FastQC.

**Code snippet for performing initial quality check on raw Fastq read files:**

$ fastqc -t 10 *.fastq.gz

$ multiqc .

where *t* is the number of threads to be used to run FastQC. The “.” in the multiqc command represents the current directory.

**C. Data cleaning and final quality check**

The sequence reads in the raw .fastq.gz files generally exhibited good quality. However, some low-quality bases and adapters are present in the sample reads, which need to be trimmed from the 3' end. After quality control and adapter trimming, the length of the reads varied widely. This variation may cause some reads to lose their mate pairs. Therefore, in addition to trimming, it is also essential to filter reads based on length (default cutoffs: TrimGalore - 20; fastp - 15).

**Figure 3.**
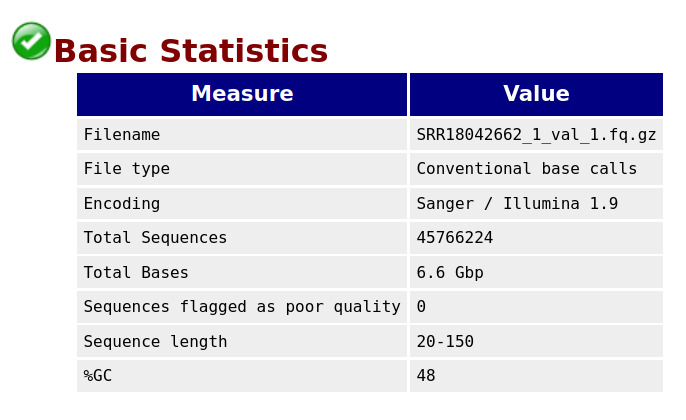
Final QC: Basic statistics for the sample SRR18042662 after adapter trimming

**Figure 4.**
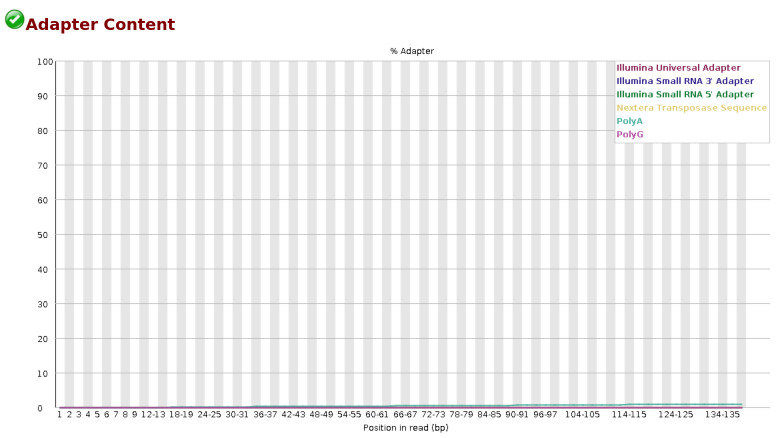
Final QC: Adapter content after trimming for sample SRR18042662

Trimming tools such as fastp (version 0.23.4) and TrimGalore can automatically detect and trim adapters. We used both tools to determine the best results. After trimming, we checked the quality of reads using FastQC. Figures 3 and 4 represent the basic statistics and adapter content of SRR18042662 sample dataset after trimming.

**Code snippet for trimming adapters using TrimGalore and Fastp:**

*# A. TrimGalore*

*# For paired-end reads*

$ trim_galore --quality 20 --stringency 7 --paired SRR18042662_1.fastq.gz SRR18042662_2.fastq.gz --output_dir 2.Trimmed_reads/ --cores 2 --retain_unpaired

*# For single-end reads*

$ trim_galore --quality 20 --stringency 7 SRR18042663.fastq.gz --output_dir 2.Trimmed_reads/ --cores 2

*# B. Fastp*

*# For paired-end reads*

$ fastp -i SRR18042662_1.fastq.gz -I SRR18042662_2.fastq.gz -o SRR18042662_1_clean.fastq.gz -O SRR18042662_2_clean.fastq.gz --detect_adapter_for_pe --cut_tail --correction --overrepresentation_analysis --json SRR18042662.fastp.json --html SRR18042662.fastp.html --thread 16

*# For single-end reads*

$ fastp -i SRR18042663.fastq.gz -o SRR18042663_clean.fastq.gz --cut_tail --overrepresentation_analysis --json SRR18042663.fastp.json --html SRR18042663.fastp.html --thread 16

TrimGalore parameters:

--quality 20: Trim bases from the ends of reads based on low Phred Score quality (< 20).

--stringency 7: Trims adapter sequences from ends only if there is an overlap of 7 or more bases with the adapter sequence.

--paired: parameter for specifying paired-end reads as input.

--retain unpaired: Removal of low-quality bases and adapters will lead to some reads having very low read lengths. These reads fail to meet length cutoffs and are removed. Their mate pairs remain as single reads and are retained in separate files.

Fastp parameters:

--detect_adapter_for_pe: Automatic adapter detection for paired-end sequences. If disabled, adapter detection will be assumed for single-end sequences.

--cut_tail: trims read at 3′ end based on low base quality scores by moving a sliding window from 5′ to 3′.

--correction: For paired-end data; read pairs are overlapped to find proper matches, and bases with low quality on one read are corrected to a high quality of their corresponding base on the other read.

--overrepresentation analysis: gives information on where the detected overrepresented sequences are mostly distributed.

When comparing the results obtained from TrimGalore and fastp, we observed that TrimGalore retained almost 99.5% of reads across all samples. Although the Q20 and Q30 Phred values improved with fastp, more reads and bases were removed after trimming (see Table 2 below). Therefore, we chose to proceed with TrimGalore as the preferred trimming tool. We then executed FastQC and MultiQC on the trimmed reads to ensure these were of high quality for downstream analysis.

**Table 2.** Read-base distribution before and after trimming using Fastp and TrimGalore

Sample	Raw Reads	Raw read bases (GB)	Fastp-trimmed reads	Fastp-trimmed bases (GB)	TrimGalore-trimmed reads	TrimGalore-trimmed bases (GB)
SRR18042662	92,046,490	13.807	88,159,790 (95.78)	12.861	91,532,448 (99.44)	13.376
SRR18042663	28,312,422	2.407	26,720,747 (94.38)	2.194	26,932,291 (95.13)	2.213
SRR18042664	31,636,161	2.689	31,455,656 (99.43)	2.669	31,612,738 (99.93)	2.680
SRR18042665	200,335,050	30.050	192,054,572 (95.87)	27.590	199,221,410 (99.44)	28.677
SRR18042666	33,538,680	2.851	33,242,144 (99.12)	2.821	33,120,616 (98.75)	2.791
SRR18042667	35,517,838	3.019	35,303,710 (99.40)	2.996	35,487,051 (99.91)	3.007
SRR18042668	121,942,300	18.291	115,871,486 (95.02)	16.789	120,703,024 (98.98)	17.522
SRR18042669	30,961,231	2.632	30,760,768 (99.35)	2.610	30,834,234 (99.59)	2.594
SRR18042670	49,600,226	4.216	49,297,844 (99.39)	4.183	49,569,155 (99.94)	4.202

*Note: In the above table, the numbers represented in parenthesis indicate the percentage of reads retained after trimming with fastp and trimgalore.*

**D. Mapping high-quality reads to reference genomes**

The next step is to map the trimmed reads to a reference genome (Human - hg38) to identify the genomic locations of all the reads. For genome mapping, there are two leading splice-aware alignment tools: STAR and HISAT2. STAR is a super-fast, highly accurate, and memory-intensive splice-aware aligner. STAR reports a high proportion of uniquely mapped reads as compared to any other topmost splice-aware alignment tools. STAR also maps the non-contiguous reads to the reference genome by a technique called soft clipping. This method improves mapping accuracy, and almost every read is mapped to the genome. However, most of the reads tend to multimap at different genomic locations. There is a chance that the shorter pathogenic reads from the dual RNA-Seq dataset will be multi-mapped to the host genome, and these may also be reported as uniquely mapped reads by soft clipping. In order to avoid such mismapping of reads, we emphasize certain critical checkpoints that are discussed in detail in the following sections of the protocol.

The following criteria are defined for mapping dual RNA-Seq data:

• A high number of uniquely mapped reads.

• A low number of multi-mapping reads.

• A higher number of unmapped reads (likely belonging to a pathogen).

Although both STAR and HISAT2 yield good results independently, we validated the above criteria by mapping our data with both tools.

1. When comparing RefSeq and Ensembl annotations, we chose to work with RefSeq, as it covers most genes with its simplest version of genome annotation [17]. Therefore, as the next step, we downloaded the reference genome assembly (hg38) and annotation file (.gtf) from NCBI RefSeq - GCF_000001405.40. Go to the link to download the reference genome of *Homo sapiens*: https://www.ncbi.nlm.nih.gov/datasets/genome/GCF_000001405.40/

2. In the above link, click the download button. Then, from the popup screen, select RefSeq only, and choose to download it.

3. Uncompress the downloaded file and collect the reference genome (.fna) and annotation files (.gtf and .gff3). Store these files in a separate folder.

**Code snippet for indexing reference genome using STAR and HISAT2:**

*# Indexing Reference Genome using STAR*

$ STAR --runThreadN 4 --runMode genomeGenerate --genomeDir STAR_index --genomeFastaFiles GCF_000001405.40_GRCh38.p14_genomic.fna --sjdbGTFfile genomic.gtf

*# Indexing Reference Genome using HISAT2*

*# Extracting Splice Sites*

$ extract_splice_sites.py genomic.gtf > splice_sites.tsv

*# Extracting Exons*

$ extract_exons.py genomic.gtf > exons.tsv

*# Building Genome Index using exon and splice site information*

$ hisat2-build GCF_000001405.40_GRCh38.p14_genomic.fna hisat2_hg38_index/hg38_index -p 40 --ss splice_sites.tsv --exon exons.tsv

STAR indexing parameters:

--runThreadN 4: Number of cores to use for processing. It is best to use a minimum number of threads/cores, either 4 or 2, as STAR runs faster, exhausting more RAM.

HISAT2 indexing parameters:

--extract_splice_sites.py: To extract and incorporate splice site information from .gtf so that transcripts do not map mostly to intronic regions and also for identifying transcript isoforms.

--extract_exons.py: To extract exon sites from .gtf, so that transcripts map exactly to exonic regions on the genome.

**Code snippet for reference genome mapping using STAR and HISAT2:**

*# Mapping to the Reference Genome using STAR*

*# Paired-end reads*

$ STAR --genomeDir STAR_index --runThreadN 2 --readFilesCommand zcat --readFilesIn SRR18042662_trimmed_1.fq.gz SRR18042662_trimmed_.fq.gz –outFileNamePrefix B.Mapping_STAR/SRR18042662__STAR --outSAMtype BAM Unsorted

*# Single-end reads*

$ STAR --genomeDir STAR_index --runThreadN 2 --readFilesCommand zcat --readFilesIn SRR18042663_trimmed.fq.gz --outFileNamePrefix B.Mapping_STAR/SRR18042663__STAR --outSAMtype BAM Unsorted

*# Mapping to the Reference Genome using HISAT2*

*# Paired-end reads*

$ hisat2 --dta -x hisat2_hg38_index/hg38_index -1 SRR18042662_trimmed_1.fq.gz -2 SRR18042662_trimmed_2.fq.gz -p20 --un-conc-gz B.Mapping_HISAT2/SRR18042662_Unmapped_pairs --summary-file B.Mapping_HISAT2/SRR18042662_align_stats.txt | samtools view -@15 -b -S | samtools sort -n -@15 -o B.Mapping_HISAT2/SRR18042662.sorted.bam -O BAM

*# Single-end reads*

$ hisat2 --dta -x hisat2_hg38_index/hg38_index -U SRR18042663_trimmed.fq.gz -p20 --un-conc-gz B.Mapping_HISAT2/SRR18042663_Unmapped_reads.fq.gz --summary-file B.Mapping_HISAT2/SRR18042663_align_stats.txt | samtools view -@15 -b -S | samtools sort -n -@15 -o B.Mapping_HISAT2/SRR18042663.sorted.bam -O BAM

The --dta parameter in HISAT2 reports the alignments as the transcript assemblers used to provide. After mapping all our samples with both STAR and HISAT2, the mapping results were tabulated in Table 3 for comparative analysis of the mapping performances.

**Table 3.** Comparative mapping analysis of HISAT2 vs. STAR

HISAT2 vs. STAR*	Total reads	Unique	Multi-mapped (should be lesser)	Unmapped (should be more for dual RNA-Seq data)
SRR18042662	45766224	31950488 (69.81%)	5220616 (11.41%)	8595120 (18.78%)
		34890020 (76.24%)*	6217589 (13.59%)*	4658615 (10.18%)*
SRR18042663	26932291	15352634 (57.00%)	6347595 (23.57%)	5232062 (19.43%)
		15427880 (57.28%)*	6761705 (25.1%)*	4742706 (17.61%)*
SRR18042664	31612738	21878890 (69.21%)	5409698 (17.11%)	4324150 (13.68%)
		22095054 (69.89%)*	5731876 (18.13%)*	3785808 (11.98%)*
SRR18042665	99610705	39819023 (39.97%)	41065084 (41.23%)	18726598 (18.80%)
		38181484 (38.33%)*	52467481 (52.67%)*	8961740 (8.99%)*
SRR18042666	33120616	21182314 (63.96%)	7473519 (22.56%)	4464783 (13.48%)
		21391317 (64.59%)*	7870514 (23.76%)*	3858785 (11.65%)*
SRR18042667	35487051	24954036 (70.32%)	5706459 (16.08%)	4826556 (13.60%)
		25238589 (71.12%)*	6073050 (17.11%)*	4175412 (11.77%)*
SRR18042668	60351512	37169324 (61.59%)	11599955 (19.22%)	11582233 (19.19%)
		40350621 (66.86%)*	14124605 (23.4%)*	5876286 (9.74%)*
SRR18042669	30834234	20287934 (65.80%)	6285903 (20.39%)	4260397 (13.82%)
		20528529 (66.58%)*	6817201 (22.11%)*	3488504 (11.31%)*
SRR18042670	49569155	37725432 (76.11%)	8155703 (16.45%)	3688020 (7.44%)
		38124908 (76.91%)*	8671102 (17.5%)*	2773145 (5.6%)*

Note: In the above table, * refers to the STAR tool, the corresponding number of reads, and percentage of reads mapped using STAR.

From Table 3, it is evident that although STAR produced more uniquely mapped reads in most samples, it is noteworthy that HISAT2 generated fewer multi-mapped reads and more unmapped reads across all samples compared to STAR. Therefore, we can use HISAT2 for mapping reads to eukaryotic genomes.

Assuming the unmapped reads belong to pathogens, we mapped these to the Mtb genome (RefSeq - GCF_000195955.2) using the BWA aligner [18] to obtain the mapping percentages shown below in Table 4 (second column).

Another alternative mapping approach is to map the adapter-trimmed reads directly to the Mtb genome first. This approach is justified because prokaryotic reads are fewer and shorter compared to eukaryotic reads. When using the previously described mapping method, there is a risk of missing some of the prokaryotic reads, as these shorter reads may misalign with the eukaryotic genome. Therefore, mapping to the Mtb genome first will increase confidence in our mapping steps (Table 4). Subsequently, we extracted the unmapped reads and aligned them to the hg38 genome using HISAT2 (Table 5).

This method was followed earlier by Espindula et al. [10], and significant changes were observed in the number of reads mapped using all possible approaches. This protocol extensively demonstrates the same pathogen-first mapping method by elaborating the steps in a more detailed manner with all the code snippets, which can be altered and used.

**Code snippet for alternative pathogen-first mapping approach:**

*# Indexing Mtb genome using BWA short-read aligner*

$ bwa index GCF_000195955.2_ASM19595v2_genomic.fna

*# Alternative Mapping Approach*

*# A. Mapping trimmed reads first to Mtb Genome using BWA.*

*# Paired-end reads*

$ bwa mem Mtb_genome/GCF_000195955.2_ASM19595v2_genomic.fna SRR18042662_trimmed_1.fq.gz SRR18042662_trimmed_2.fq.gz -t 20 | samtools view -@15 -b -S | samtools sort -n -@15 -o B.Mapping_BWA_PathogenFirst/SRR18042662.sorted.bam -O BAM

*# Single-end reads*

$ bwa mem Mtb_genome/GCF_000195955.2_ASM19595v2_genomic.fna SRR18042663_trimmed.fq.gz -t 20 | samtools view -@15 -b -S | samtools sort -n -@15 -o B.Mapping_BWA_PathogenFirst/SRR18042663.sorted.bam -O BAM

*# B. Extract unmapped reads using samtools*

$ samtools view -b -f 4 B.Mapping_BWA_PathogenFirst/SRR18042662.sorted.bam > B.Mapping_BWA_PathogenFirst/SRR18042662_unmapped.sorted.bam

*# C. Convert sorted bam files to fastq files (paired and single-end)*

$ bedtools bamtofastq -i SRR18042662_unmapped.sorted.bam -fq SRR18042662_unmapped_Host_1.fq -fq2 SRR18042662_unmapped_Host_2.fq

$ samtools fastq SRR18042663_unmapped.sorted.bam > SRR18042662_unmapped_Host.fq

*# D. Map the unmapped (Host-reads) to hg38 using HISAT2*

*# Refer HISAT2 mapping command (paired and single-end) from previous snippets.*

This way of mapping the reads will result in appropriately aligned prokaryotic reads so that we can more accurately use them to study gene expression levels of both the pathogen and the host.

**Table 4.** Comparison of mapping between pathogen-first and unmapped Mtb reads (from HISAT2)

Sample	No. of unmapped reads (pathogen) from HISAT2 results mapped to Mtb genome	No. of adapter-trimmed reads mapped to Mtb (pathogen-first method)
SRR18042662 (PE)	4448104 (25.88%)	4715080 (5.15%)
SRR18042663	3859948 (73.77%)	3859968 (14.33%)
SRR18042664	2251961 (52.08%)	2252019 (7.12%)
SRR18042665 (PE)	10709925 (28.60%)	12181384 (6.11%)
SRR18042666	1831236 (41.02%)	1831276 (5.53%)
SRR18042667	2433725 (50.42%)	2433770 (6.86%)
SRR18042668 (PE)	6380085 (27.54%)	6883877 (5.70%)
SRR18042669	1514060 (35.54%)	1514089 (4.91%)
SRR18042670	1507197 (40.87%)	1507299 (3.04%)

From Table 4, we can visualize the significant increase of ~0.1–0.5 million in mapping numbers in the case of paired-end reads, whereas for single-end reads, there is an increase of around 100 reads. Some of the reads may not map in pairs in the case of paired-end reads. Hence, the mapping percentages are lower in paired-end reads compared to single-end reads. The mapping percentages are even lower in the pathogen-first mapping approach since we are using the complete host–pathogen trimmed reads directly for mapping to the Mtb genome.

**Table 5.** Comparison of mapping between host-first and unmapped host reads (from BWA)

Sample	No. of reads mapped to hg38 (host-first approach)			No. of unmapped reads (host) mapped to hg38 from BWA results (pathogen-first approach)		
	**Unique**	**Multi-mapped**	**Unmapped**	**Unique**	**Multi-mapped**	**Unmapped**
SRR18042662	31950488 (69.81%)	5220616 (11.41%)	8595120 (18.78%)	31893407 (69.68%)	5144240 (11.24%)	6320738 (13.81%)
SRR18042663	15352634 (57.00%)	6347595 (23.57%)	5232062 (19.43%)	15352703 (57.00%)	6347634 (23.56%)	1371986 (5.09%)
SRR18042664	21878890 (69.21%)	5409698 (17.11%)	4324150 (13.68%)	21879065 (69.20%)	5409463 (17.11%)	2072191 (6.55%)
SRR18042665	39819023 (39.97%)	41065084 (41.23%)	18726598 (18.80%)	39652369 (39.80%)	40503121 (40.66%)	13305107 (13.35%)
SRR18042666	21182314 (63.96%)	7473519 (22.56%)	4464783 (13.48%)	21182318 (63.95%)	7473434 (22.56%)	2633588 (7.95%)
SRR18042667	24954036 (70.32%)	5706459 (16.08%)	4826556 (13.60%)	24954231 (70.31%)	5706225 (16.07%)	2392825 (6.74%)
SRR18042668	37169324 (61.59%)	11599955 (19.22%)	11582233 (19.19%)	37076766 (61.43%)	11444024 (18.96%)	8361031 (13.85%)
SRR18042669	20287934 (65.80%)	6285903 (20.39%)	4260397 (13.82%)	20287963 (65.79%)	6285867 (20.38%)	2746315 (8.90%)
SRR18042670	37725432 (76.11%)	8155703 (16.45%)	3688020 (7.44%)	37725559 (76.10%)	8155417 (16.45%)	2180880 (4.39%)

In Table 5, we compare the mapping results of unmapped reads extracted from the BWA results with HISAT2 mapping results from Table 3. We observe that the unmapped reads from the pathogen-first approach have a good number of uniquely mapped reads and is marginally increased for single-end reads. The number of multi-mapped reads is also marginally lower compared to the host-first mapping approach. Unmapped reads are minimal, as we initially mapped the reads to the Mtb genome. Though a significant increase/decrease in the number of host reads may not be observed during comparison, the host reads from the pathogen-first mapping approach are still found to have impactful results while performing downstream analysis.

Therefore, we consider approach B (pathogen-first mapping) as the best approach and HISAT2 for mapping to the eukaryotic genome when working with Dual RNA-Seq data.

*Note: There may still be some reads left unmapped after mapping to genomes of both the host and the pathogen. There may even be a possibility of occurrence of horizontal gene transfer (HGT) events, wherein, other bacteria or viruses other than the pathogen of interest may have invaded the host cells. In order to check this, users may extract only the unmapped reads and map these reads against genomes of other species using BLAST search.*

**E. Read count quantification using featureCounts**

In the previous section, we mapped all the reads to their respective genomes. The next step is to count the number of reads to determine which gene or exonic region each read has mapped to the genome. Therefore, we performed a quantification step for both the pathogen-mapped and host-mapped reads using the RefSeq annotation files (.gtf) of both Mtb and hg38. The featureCounts tool was used to count reads that are mapped to exonic regions and genes.

**Code snippet for quantification of read counts:**

*# Read Count Quantification: FeatureCounts - Pathogen read counting*

*# Paired-end reads*

$ featureCounts -a Mtb_Genome/genomic.gff -t 'gene' -g 'Name' SRR18042662.sorted.bam SRR18042665.sorted.bam SRR18042668.sorted.bam -p -o C.Read_Count_Quantification/Pathogen_Quant_PE_Togene_genename.tsv -O --countReadPairs -T 40

*# Single-end reads*

$ featureCounts -a Mtb_Genome/genomic.gff -t 'gene' -g 'Name' SRR18042663.sorted.bam SRR18042664.sorted.bam SRR18042666.sorted.bam SRR18042667.sorted.bam SRR18042669.sorted.bam SRR18042670.sorted.bam -o C.Read_Count_Quantification/Pathogen_Quant_SE_Togene_genename.tsv -O -T 40

*# Merge .tsv files from both paired-end and single-end quantification results.*

$ cut -f1,7- Pathogen_Quant_PE_Togene_genename.tsv | grep -v "#" > temp_Pathogen_Quant_PE.tsv

$ cut -f1,7- Pathogen_Quant_SE_Togene_genename.tsv | grep -v "#" > temp_Pathogen_Quant_SE.tsv

$ join -o auto -e '0' -a 1 -a 2 -1 1 -2 1 temp_Pathogen_Quant_PE.tsv temp_Pathogen_Quant_SE.tsv > Pathogen_Quantification_matrix.tsv

*# Read Count Quantification: FeatureCounts - Host read counting*

*# For paired-end and single-end reads (Host), follow the above code snippets by replacing "genomic.gff" file with RefSeq hg38 annotation file (.gff3)*

featureCounts parameters:

-t ‘gene’: reads will be mapped to the “gene” feature from the annotation file. Default: ‘exon’.

-g ‘Name’: the quantified reads will be grouped under gene names from .gff if “Name” is mentioned. Default: ‘gene_id’.

-p: To specify that the reads are paired-end

-O: A parameter for quantifying reads with minimum overlapping bases also.

--countReadPairs: By mentioning this parameter, fragments will be quantified instead of reads. This is applicable for paired-end reads.

Join command parameters:

-o auto: A parameter to apply a simple format to the output file.

-e ‘0’: Parameter to fill empty values with zero.

-a: Parameter to fill the file numbers.

-1 1: Join files based on column 1 for file 1.

-2 1: Join files based on column 1 for file 2.

The read count results obtained from the previous step can be used to infer the genes that are differentially expressed under different treatment conditions.

## Validation of protocol

The number of reads retained after each step in the analysis significantly impacts data quality, especially when dealing with a dataset containing reads from multiple species. In this protocol, we have identified and listed the top-performing software predominantly used for such analyses. Additionally, we have tabulated the results obtained at each step after thoroughly benchmarking these software tools. Besides the software chosen for trimming and mapping in this protocol, the other sections were validated in earlier studies, which include mapping strategy [10] and choice of genome annotation [17].

## General notes and troubleshooting

**General notes**

1. In this protocol, we have detailed the quality control, mapping, and quantification of read counts specific to both host and pathogen, as these are the most crucial steps in generating highly confident data for downstream analysis. The read count results from these steps are obtained after proper validation at each stage of analysis.

2. We are not demonstrating further downstream analysis in detail here, as it would be a repetition of *Bio-protocol* references [19–22], and the statistical preferences vary widely based on individual research perspectives.

3. The mapping strategies discussed are of vital importance. By using a pathogen-first approach, we ensure higher confidence in mapping steps, particularly by reducing misalignment issues with shorter prokaryotic reads. This strategy also helps in accurately identifying pathogen reads prior to mapping the remaining reads to the host genome using HISAT2. Such meticulous mapping strategies are critical for obtaining reliable data for efficient downstream analyses.

4. The raw read counts obtained from quantification can be further normalized using the DESeq2 package in R, which applies the median of ratios method of normalization. This method accounts for sequencing depth and RNA composition without considering gene length, as differential expression (DE) analysis compares read counts between sample groups for the same gene. The calculation of median ratios and the script used to obtain normalized counts are also available online at https://hbctraining.github.io/DGE_workshop/lessons/02_DGE_count_normalization.html.

5. DESeq2 works with sample replicates and tests how the variances calculated from read count data of replicates are dispersed. There are several methods for estimating the dispersion based on the nature of the dataset. Generally, in case of fewer replicates, the data may get adjusted to fit even the outliers into the dispersion model; this in turn affects the downstream analysis and biases the gene expression levels. Therefore, it is advisable to have more replicate samples so that the accuracy of data is maintained and the outliers are easily removed. DESeq2 uses negative binomial distribution by default, which is the most widely used dispersion method, as it is extensively designed for biological systems exhibiting excessive variability. Whenever there are unequal numbers of replicates, generalized linear models (GLMs) can be used, as they are flexible to model unequal variances.

6. In the case of the dataset having non-replicate samples, edgeR can be used to perform differential expression analysis:

**Code snippet for DEG analysis using edgeR:**

*# Install and load edgeR library and count databases*

*install.packages(“edgeR”)*

*library(edgeR)*

*readcount <- read.table(“count_data.tsv”, header=TRUE, row.names=1)*

*# Create DEG object*

*dds <- DGEList(counts=readcount, group=c(“control”,”treatment”))*

*# The above line creates a DEG object using the readcounts of one control and one treatment sample.*

*# Data normalization*

*dds <- calcNormFactors(dds, method=”TMM”)*

*# Data normalization using Trimmed means of M-values method.*

*# Estimation of Dispersion*

*dds <- estimateDisp(dds, robust=TRUE)*

*# Fitting negative binomial model*

*fit <- glmFit(dds, design=model.matrix(~group))*

*# DEG analysis*

*deg <- glmLRT(fit, coef=2)*

*# Extracting significantly expressed genes based on p-value cutoffs*

*deg_results <- deg[deg$table$PValue < 0.05, ]*

7. After normalization, differential gene (DE) expression analysis can be performed by comparing treatment and control groups from the sample dataset. The DE analysis results can be validated by appropriate statistical tests (Wald test, DESeq2), with significantly expressed genes marked by p-values, false discovery rate (FDR), and log-fold changes. The steps for performing DE analysis are described in the *Bio-protocol* by Hoerth et al. [23] and Fernández et al. [24].

8. After analyzing gene expression levels across several conditions, significantly upregulated and downregulated genes can be functionally annotated using homology search by BLAST.

9. Gene ontology analysis can be performed using BiNGO, a Cytoscape-based plugin, discussed in detail by Duarte et al. [22]. The same method can be followed to predict the functional role of differentially expressed genes. As an alternative, CytoHubba, another cytoscape plugin, can also be used for this purpose. This tool ranks the topmost hub genes out of the significantly expressed genes.

10. Pathway enrichment of differentially expressed genes can also be performed using online tools like DAVID, as mentioned by Chemello et al. [20]. Graphite Web [25] is another resource for pathway enrichment analysis.

**Troubleshooting**

1. The software and datasets section of the manuscript represents the corresponding latest version numbers, dated as of while performing this analysis. Therefore, it is always recommended that you install and use the latest version of the software with all the bug fixes.

2. While installing software using conda, there are possibilities for already existing versions of the software to be upgraded/downgraded automatically in order to manage package dependencies. Some tools like trimgalore require FastQC and cutadapt to be installed separately, as version compatibility conflicts could arise. In such cases, users can still try to switch the order of software installation to have the latest version in usage.

3. After trimming, it is essential to check whether low-quality bases and adapters are completely removed from the raw reads. The FastQC reports after trimming need to be checked for residual adapter content that may be present. In some cases, the poly-A tail and other bases can still be found. In this case, raw data can be again subjected to trimming using trimgalore by refining the parameters for adapter trimming.

4. While extracting the unmapped reads from .bam files, it is important to ensure that the reads are properly fetched as paired-end, without missing out any reads because of the absence of mapping pairs. In order to address this, the .bam files used for extracting the reads need to be properly sorted by “name” by using “samtools sort -n“ and not by “coordinates”.

**GitHub page links for the above-mentioned software:**

1. TrimGalore: https://github.com/FelixKrueger/TrimGalore

2. SAMtools: https://github.com/samtools/samtools?tab=readme-ov-file
